# Molecular characterization of juvenile fish from the Amazon estuary using DNA barcoding approach

**DOI:** 10.1371/journal.pone.0292232

**Published:** 2023-09-28

**Authors:** Ítalo Lutz, Thais Martins, Felipe Araújo, Charles Ferreira, Paula Santana, Josy Miranda, Suane Matos, Jefferson Sousa, Luciano Pereira, Bianca Bentes, Raimundo da Silva, Ivana Veneza, Iracilda Sampaio, Marcelo Vallinoto, Grazielle Evangelista Gomes

**Affiliations:** 1 Laboratório de Genética Aplicada, Instituto de Estudos Costeiros, Universidade Federal do Pará, Bragança, Pará, Brazil; 2 Coordenação de Zoologia, Museu Paraense Emílio Goeldi, Belém, Pará, Brazil; 3 Núcleo de Ecologia Aquática e Pesca da Amazônia, Universidade Federal do Pará, Belém, Pará, Brazil; 4 Universidade Federal do Oeste do Pará, Monte Alegre, Pará, Brazil; 5 Laboratório de Evolução, Instituto de Estudos Costeiros, Universidade Federal do Pará, Bragança, Pará, Brazil; Laboratoire de Biologie du Développement de Villefranche-sur-Mer, FRANCE

## Abstract

The efficiency of the DNA barcoding relies on sequencing fragment of the Cytochrome C Subunit I (COI) gene, which has been claimed as a tool to biodiversity identification from distinct groups. Accordingly, the goal of this study was to identify juvenile fish species along an estuary of Caeté River in the Brazilian Blue Amazon based on. For this purpose, we applied the DNA barcoding and discuss this approach as a tool for discrimination of species in early ontogenetic stages. A 500-bp fragment was obtained from 74 individuals, belonging to 23 species, 20 genera, 13 families and seven orders. About 70% of the 46 haplotypes revealed congruence between morphological and molecular species identification, while 8% of them failed in identification of taxa and 22% demonstrated morphological misidentification. These results proved that COI fragments were effective to diagnose fish species at early life stages, allowing identifying all samples to a species-specific status, except for some taxa whose COI sequences remain unavailable in public databases. Therefore, we recommend the incorporation of DNA barcoding to provide additional support to traditional identification, especially in morphologically controversial groups. In addition, periodic updates and comparative analyses in public COI datasets are encouraged.

## Introduction

Fishes represent more than half of all vertebrates, comprising more than 30,000 described species worldwide many of them representing important economic resources [1, 2]. Therefore, a correct identification of taxa is essential to design proper managements for both fish conservation and fisheries, besides being useful to the authenticity of processed food products and drugs derived from fish sources [3, 4].

On the other hand, unequivocal diagnosis of species remains a challenging task since some species present deep morphological ontogenetic changes, remarkable sexual dimorphism or even habitat-related anatomical adaptations. Moreover, several species share similar morphological characters (i.e. cryptic species), hindering their discrimination based on traditional morphology, eventually leading to ambiguities and inconsistencies [5–7].

As a strategy to overcome the limitations of morphology-based taxonomy the DNA barcoding initiative has been launched to provide a reliable system of molecular identification of species in both vertebrates and invertebrates [8]. In fact, this approach has been remarkably accurate to identify organisms at species level [9–12]. By sequencing a fragment of nearly 650 pb close to 5’ region of the mitochondrial Cytochrome C Oxidase Subunit I (COI) gene, referred to as DNA Barcode, it has been possible to assess the similarities within COI fragments among different species available in public databases, such as the National Center for Biotechnology Information (NCBI) GenBank (http://www.ncbi.nlm.nih.gov) [13] and the BOLD Platform (Barcoding of Life Database - http://www.barcodinglife.org) [14]. In addition, applying species delimitation algorithms in different organisms enhances the precise identification of species [15], even those characterized by high morphological similarities [16, 17], providing extra validation for taxonomical studies. Accordingly, the efficacy of DNA barcoding in identifying fish species independently on their development stages has been confirmed, resulting in new data about local diversity, disambiguation in species identification and efficient conservation policies [7, 18–21].

Estuarine environments are habitats characterized by increased primary production and consequently encompass high levels of biodiversity [22]. In fact, these areas are widely used as breeding sites, nursery, juvenile development, feeding and shelter for different fish species [23, 24]. The Caeté River estuary (approximately 200 km^2^), is located within one of the largest mangrove regions worldwide, the Amazonian Macrotidal Mangrove Coast (7.591.09 km^2^) in northern Brazil [25, 26]. This estuary follows a semidiurnal tidal regime, with a mean amplitude of 3.3 m, a warm and humid climate, an average temperature of 25°C and rainfall index above 2,000 mm [26, 27].

The ichthyofauna of Caeté River includes a high number of species adapted to continuous salinity variations [28]. Morphological studies in this estuary placed Sciaenidae, Engraulidae, Gobiidae, Tetraodontidae, Ariidae, Aspredinidae, Carangidae and Haemulidae as the most abundant families. Many representatives from such families complete their life cycle within the estuarine system while some others exploit the area only during reproductive periods [23, 28, 29]. In a faunistic inventory of fish larvae, 63 taxa were identified in Caeté estuary [30], but many specimens could not be identified at species level. This may occur because individuals at early development stages usually show distinct phenotypic traits in relation to adults and hence identifications based exclusively on morphological data can be unsuccessful or lead to misidentifications [9, 31, 32]. Furthermore, during initial stages of development, specimens from different fish species present some degree of morphological similarities, as commonly reported among species of Sciaenidae and Centropomidae [33].

Therefore, considering the ecological importance of the Caeté River estuary to the blue Amazon fish community at different ontogenetic stages, this study aimed to characterize and identify juvenile fish specimens at species level based on DNA barcoding under a comparative analysis with traditional morphological identification. We then discuss the use of the DNA barcodes in association with classical identification as an integrative approach to overcome the obstacles of recognizing the diversity of juvenile ichthyofauna in a biodiversity hotspot.

## Materials and methods

### Ethics statement

The collection of samples were carried out along the Caeté River estuary, which is part of the Caeté-Taperaçu Marine Extractive Reserve, in northern Brazil after authorization by the Instituto Chico Mendes de Conservação da Biodiversidade (ICMBio), under licensing on behalf of Bianca Bentes da Silva (license number SISBIO 47679–1). After collection, the biological tissue sample was obtained and specimens were euthanized in a solution of 0.20 mL clove oil [34], and fixed in 10% formalin for at least 48 hours before being transferred to 70% ethanol for posterior morphological comparations.

### Study area, sampling and tissue banks

**Fig 1:**
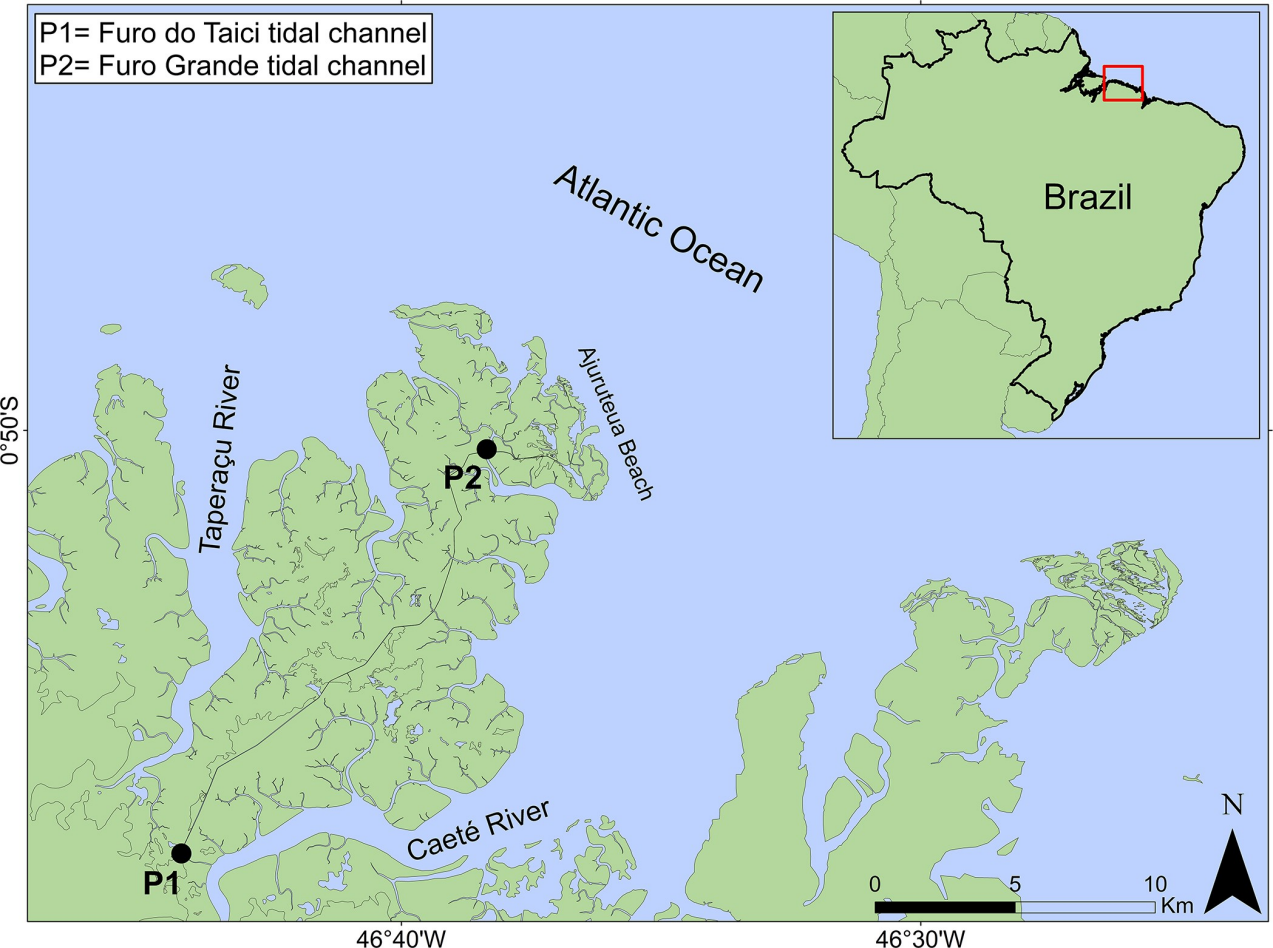
Map of Caeté River estuary. Indication of the two collection sites of fish samples: Furo do Taici and Furo Grande, Bragança–PA, Brazilian Blue Amazon.

Individuals were captured during expeditions carried out by Projeto Meros do Brasil along the Caeté River estuary, in Northeastern region of the state of Pará. This region is located on the Brazilian northern coast, which corresponds to the Blue Amazon (Fig 1). In this area, we selected two collection sites in order to sample the highest number in species diversity, taking into account the horizontal distribution of organisms as influenced by salinity. The collection site 1 (Furo do Taici, 0°58’08.4"S 46°44’15.6"W) is closest to the Caeté River, being characterized by low salinity levels in contrast to site 2 (Furo Grande, 0°50’26.0"S 46°38’21.8"W), which is a tidal channel presenting high levels of salinity [28, 35].

We conducted monthly collections over a year, starting from October 2012 to October 2013. The expeditions were carried out during lunar tides for 12 consecutive hours, using a block net (30 m long and 4 m high, with a mesh opening of 20 mm). These nets were placed during the first low tide of the day. The collections were performed by the team of the Laboratório de Bioecologia Pesqueira (LABIP) from the Instituto de Estudos Costeiros (IECOS) at Universidade Federal do Pará (UFPA), in association with the staff from the project “Meros do Brasil”. Right after sample processing and biometric analyses, a minimum of two individuals of each collected species was identified using molecular methods. All individuals investigated here were considered juveniles according to their ontogenetic development and total length.

Firstly, the samples were identified based on specific literature [1, 33, 36]. Afterwards, a biological sample (fins) from each specimen was taken, stored in 90% ethanol under refrigeration until molecular procedures. The tissue samples were deposited at the tissue bank of the Laboratório de Genética Aplicada (LAGA) from the Instituto de Estudos Costeiros (IECOS) at Universidade Federal do Pará (UFPA).

### Laboratory procedures

The DNA was isolated using a commercial kit (Wizard Genomic®, Promega), following the manufacturer’s instructions. The DNA products were added to a 2L Gel red™ solution and submitted to electrophoresis in a 1% agarose gel, under 60V for approximately 40 minutes to check the quality of DNA samples.

The high-quality isolated DNA products were used to amplify the barcode COI portion via Polymerase Chain Reaction (PCR) using the primers: COI FishF1 – 5’ - TCAACCAACCACAAAGACATTGGCAC – 3’; COI FishF2 – 5’ - TCGACTAATCATAAAGATATCGGCAC – 3’; COI FishR1 – 5’ - TAGACTTCTGGGTGGCCAAAGAATCA – 3’; COI FishR2 – 5’ - ACTTCAGGGTGACCGAAGAATCAGAA - 3’ [9].

The PCRs comprised 1.5 μL of buffer (10x); 2.4 μL of dNTPs (1.25 mM); 0.6 μL of MgCl_2_ (50 mM); 0.6 μL of each primer (50 ng/μL); 1 μL of template DNA; 0.1 μL of Taq DNA polymerase (5 U/μL) and ultrapure water to a final volume of 15 μL. The amplification conditions were: initial denaturation at 94°C for 3 minutes, followed by 35 cycles with denaturation at 94°C for 40 seconds, annealing (46°C to 58°C) for 40 seconds, extension at 72°C for 40 seconds, plus a final step extension at 72°C for 4 minutes.

The amplified products were purified in PEG 8000 (polyethylene glycol) [37], and submitted to dideoxy-terminal sequencing reaction [38], using the reagents of Big Dye 3.1 kit (ABI Prism TM Dye Terminator Cycle Sequencing Ready Reaction—PE Thermo Fisher), in accordance with the manufacturer’s protocol. Subsequently, the final precipitated product was sequenced through capillary electrophoresis in an ABI 3500 XL automatic sequencer (Thermo Fisher).

### Genetic analysis

Right after sequencing, the electropherograms were visualized in the software BioEdit v. 7.2.5 [39], where the nucleotides were inspected and edited in case of possible sequencing errors. The aligned sequences comprised 74 sequences from the juvenile fish samples. The automatic alignment of the corrected sequence dataset was performed in the software Clustal X [40], implemented on BioEdit v. 7.2.5 [39]. The database was analyzed in the software DNAsp [41] to identify the haplotypes and their respective frequencies in order to speed up the molecular identification process.

The identified haplotypes were submitted to public databases of DNA sequences, such as the GenBank (National Center for Biotechnology Information - http://www.ncbi.nlm.nih.gov) [13], in which search tools are available to compare sequences like BLAST (Basic Local Alignment Search Tool) and nucleotide device Nucleotide BLAST (BLAST-N), and the BOLD platform (Barcoding of Life Database - http://www.barcodinglife.org) [14]. To facilitate the molecular identification of sampled taxa, we added sequences from public platforms and sequences of adult specimens available from the tissue bank of the Laboratory of Applied Genetics: *Micropogonias furnieri* (code 04F122) and *Macrodon ancylodon* (code 04F89).

After a comparative evaluation among haplotypes in the online databases, 31 reference sequences from public databases were added to our dataset to assist in sample identification. Therefore, the final database contained 105 sequences, used in further analyses. We performed a phylogenetic analysis in this dataset according to the following methods: i) Neighbor-Joining (NJ), ii) Maximum Likelihood (ML) and iii) Bayesian Inference (BI). The best nucleotide substitution models to the phylogenetic analysis based on BI and ML were determined by jModelTest2 [42]. In the case of NJ tree, we adopted the Kimura-2-parameter (K2P) evolutionary model [43], being the significance of the clusters based on 1000 pseudoreplicates of bootstrap [44] using the software MEGA X v10.0.5 [45].

Considering the topology recovered by NJ, we established the mean values of genetic divergence, assuming that each group would represent a unique species, adopting a corrected distance (K2P model), also in the software MEGA X v10.0.5 [45]. In addition, forensic informative polymorphic sites, species-specific mutations as well as possible stop codons in the sequenced region were checked in the same program. The sum of genetic distance and the barcode gap were estimated by the BOLD Workbench [46].

The BI analyses was performed on BEAST v. 1.10.4 [47, 48]. The runs were based on the Hasegawa–Kishino–Yano (HKY) + I + G evolutionary model, using the strict clock and Yule speciation process as tree prior. The posteriori probability was estimated with 70 million generations, sampled each 10 million steps using a burn-in of 10%. The chain convergence was visualized using the software Tracer v1.7 [49], where adequate chains were those characterized by Effective Sampling Size (ESS) >200. After the inspection, the trees were summarized on TreeAnnotator v1.10.4 [50], visualized on FigTree, v1.4.4 [51] and edited in the software Inkscape v0.92.4 (available at: https://www.inkscape.org).

Three methods of species delimitation were also performed: a) Generalized Mixed Yule Coalescent, GMYC [16] b) Automatic Barcode Gap Discovery, ABGD [52] and c) Bayesian Poisson Tree Process, PTP [17], therefore including algorithms based on either coalescent or genetic distance approaches. The coalescent approach, GMYC, was performed on Splits package [53] implemented on R v. 3.2 [54], using the pattern of a single threshold, based on BI-based tree recovered on BEAST.

The distance-based method, ABGD, was carried out using the online platform: http://www.abi.snv.jussieu.fr/public/abgd. This approach takes into account the genetic distance among taxa, considering that the highest values of intraspecific distance shall be lower than the distance values among species, characterizing the barcode gap [52]. We considered the following parameters: P min = 0.001; P max = 0.1; steps = 10; NBins = 20, relative gap = 1.0 and Kimura´s distance model [43].

The PTP is also based on coalescent theory. Using the software RAxML v.8.29, an input file was built to perform the ML analysis, following using the GTR GAMMA evolutionary model and 1000 pseudoreplicates. This methos was run online at https://species.h-its.org/ptp/, adopting the MCMC generations = 500000, thinning = 500, while other parameters were kept as default.

## Results

We isolated a fragment of about 500 bp of the barcode COI region from 74 juvenile fish samples from Caeté River estuary. Preliminary morphological identification recorded 23 species, corresponding to seven orders, 13 families and 20 genera, while the 253 polymorphic sites resulted in 46 haplotypes (Table 1). Insertions, deletions and/or stop codons were absente, indicating that the analyzed sequences corresponded to functional COI fragments. All sequences were stored in GenBank under accession numbers OQ075612 to OQ075657. In BOLD, we created the project “Rio Caete” project whose access sequence numbers range from CAETE001-20 to CAETE046-20.

**Table 1:** Comparative list of haplotypes from juvenile fish fauna of Caeté River, Bragança-PA, Blue Amazon, and public databases. BOLD = Barcode of Life Data System; GB = GenBank–National Center for Biotechnology Information; NI* = Not Identified.

Code/Haplotype	Morphological identification	BOLD/GB deposit accession number	Identified species BOLD/GB	Family	Similarity BOLD	ID Sequence/BIN Bold	Similarity GB	Access GB	Morphological identification errors
Ljo21—Hp1	*Lutjanus jocu*	CAETE001-20/OQ075612	*Lutjanus jocu*	Lutjanidae	98.82%	BZLWA400/AAA5843	99.58%	JQ842569	Not
Ljo22- Hp2	*Lutjanus jocu*	CAETE002-20/OQ075613	*Lutjanus jocu*	Lutjanidae	100%	BZLWA400/AAA5843	100%	JQ842569	Not
Aan133- Hp3	*Anableps anableps*	CAETE003-20/OQ075614	*Anableps anableps*	Anablepidae	100%	Private data	99%	LC154806	Not
Aan134- Hp4	*Anableps anableps*	CAETE004-20/OQ075615	*Anableps anableps*	Anablepidae	100%	ITAPE307/ADC4239	99%	LC154806	Not
Csp29- Hp5	*Catharops spixii*	CAETE005-20/OQ075616	*Cathorops spixii*	Ariidae	100%	Private data	98.82%	JX124751	Not
Csp32- Hp6	*Catharops spixii*	CAETE006-20/OQ075617	*Cathorops spixii*	Ariidae	99.8%	Private data	98.82%	JX124751	Not
Csp33- Hp7	*Catharops spixii*	CAETE007-20/OQ075618	*Cathorops spixii*	Ariidae	100%	Private data	99.02%	JX124751	Not
She62- Hp8	*Sciades herzbergii*	CAETE008-20/OQ075619	*Sciades couma/* NI*	Ariidae	100%	ITAPE023/ACR8601	No data	No data	**Yes**
Sti138- Hp9	*Strongylura timucu*	CAETE009-20/OQ075620	*Strongylura timucu*	Belonidae	99.79%	BAHIA172/AAE2157	99.58%	JQ365591	Not
Cpe123- Hp10	*Centropomus pectinatus*	CAETE010-20/OQ075621	*Centropomus undecimalis*	Centropomidae	100%	LIDM1014/AAC7122	99.79%	JQ365276	**Yes**
Cpe124- Hp11	*Centropomus pectinatus*	CAETE011-20/OQ075622	*Centropomus undecimalis*	Centropomidae	100%	LIDM1014/AAC7122	100%	JQ365276	**Yes**
Acl118- Hp12	*Anchovia clupeoides*	CAETE012-20/OQ075623	*Pterengraulis atherinoides*	Engraulidae	99.15%	TZGAA023/AAE8469	No data	No data	**Yes**
Asp54- Hp13	*Anchoa spinifer*	CAETE013-20/OQ075624	C*etengraulis edentulus/ Anchoa sp*.	Engraulidae	100%	Private data	99.79%	JQ398441	NI*
Asp56- Hp14	*Anchoa spinifer*	CAETE014-20/OQ075625	No data	Engraulidae	No data	No data	No data	No data	NI*
Ram144- Hp15	*Rhinosardinia amazonica*	CAETE015-20/OQ075626	No data	Clupeidae	No data	No data	No data	No data	NI*
Ram145- Hp16	*Rhinosardinia amazonica*	CAETE016-20/OQ075627	No data	Clupeidae	No data	No data	No data	No data	NI*
Cfa64- Hp17	*Chaetodipterus faber*	CAETE017-20/OQ075628	*Chaetodipterus faber*	Ephippidae	100%	MEFM126/AAB8805	100%	MH378680	Not
Dau128- Hp18	*Diapterus auratus*	CAETE018-20/OQ075629	*Diapterus auratus*	Gerreidae	99.79%	SMSA261/AAO1630	99.79%	JQ842439	Not
Dau129- Hp19	*Diapterus auratus*	CAETE019-20/OQ075630	*Diapterus auratus*	Gerreidae	100%	SMSA261/AAO1630	100%	JQ842439	Not
Dau131- Hp20	*Diapterus auratus*	CAETE020-20/OQ075631	*Diapterus rhombeus*	Gerreidae	100%	MXIV288/AAF5627	100%	JQ365329	**Yes**
Glu97- Hp21	*Genyatremus luteus*	CAETE021-20/OQ075632	*Genyatremus luteus*	Haemulidae	100%	ANGBF4391/ACC0598	100%	HQ676760	Not
Glu126- Hp22	*Genyatremus luteus*	CAETE022-20/OQ075633	*Genyatremus luteus*	Haemulidae	99.58%	ANGBF4391/ACC0598	99.79%	HQ676760	Not
Mcu172- Hp23	*Mugil curema*	CAETE023-20/OQ075634	*Mugil rubrioculus/ Mugil curema* and *Mugil rubrioculus*	Mugilidae	99.79%	MEFM108/AAA7840	99.79% e 99.37%	GU225396 and JX185212	Not
Mcu177- Hp24	*Mugil curema*	CAETE024-20/OQ075635	*Mugil rubrioculus/ Mugil curema* and *Mugil rubrioculus*	Mugilidae	100%	GBGC9240/AAA7840	99.58% e 99.15%	GU225396 and JX185212	Not
Mho176- Hp25	*Mugil hospes*	CAETE025-20/OQ075636	*Mugil hospes*	Mugilidae	100%	MFSP918/AAZ7695	100%	JQ365444	Not
Mho175- Hp26	*Mugil hospes*	CAETE026-20/OQ075637	*Mugil hospes*	Mugilidae	99.79%	MFSP918/AAZ7695	99.79%	JQ365444	Not
Msp140- Hp27	*Mugil sp*.	CAETE027-20/OQ075638	*Mugil rubrioculus/ Mugil curema* and *Mugil rubrioculus*	Mugilidae	99.79%	GBGCA5934/AAA7840	99.37% e 98.94%	GU225396 and JX185212	Not
Msp141- Hp28	*Mugil sp*.	CAETE028-20/OQ075639	*Mugil rubrioculus/ Mugil curema* and *Mugil rubrioculus*	Mugilidae	99.79%	GBGCA5934/AAA7840	99.79% e 99.37%	GU225396 and JX185212	Not
Msp142- Hp29	*Mugil sp*.	CAETE029-20/OQ075640	*Mugil rubrioculus/ Mugil curema* and *Mugil rubrioculus*	Mugilidae	99.79%	GBGCA5934/AAA7840	99.37% e 98.94%	GU225396 and JX185212	Not
Bro154- Hp30	*Bairdiella ronchus*	CAETE030-20/OQ075641	*Cynoscion acoupa*	Sciaenidae	100%	MFSP433/AAI9474	100%	JQ365312	**Yes**
Cac152- Hp31	*Cynoscion acoupa*	CAETE031-20/OQ075642	*Cynoscion acoupa*	Sciaenidae	99.79%	MFSP433/AAI9474	99.79%	JQ365312	Not
Cac162- Hp32	*Cynoscion acoupa*	CAETE032-20/OQ075643	*Cynoscion acoupa*	Sciaenidae	100%	MFSP433/AAI9474	99.79%	JQ365312	Not
Man46- Hp33	*Macrodon ancylodon*	CAETE033-20/OQ075644	*Cynoscion leiarchus*	Sciaenidae	99.79%	MFSP1986/AAE2985	99.79%	KF929804	**Yes**
Man47- Hp34	*Macrodon ancylodon*	CAETE034-20/OQ075645	*Cynoscion leiarchus*	Sciaenidae	99.58%	MFSP1986/AAE2985	99.58%	KF929804	**Yes**
Mfu159- Hp35	*Micropogonias furnieri*	CAETE035-20/OQ075646	*Bairdiella ronchus*	Sciaenidae	99.79%	ANGBF30185/ADQ8002	99.79%	KJ907229	**Yes**
Smi170- Hp36	*Stellifer microps*	CAETE036-20/OQ075647	*Stellifer microps*	Sciaenidae	100%	ANGBF30642/ADC2628	100%	KJ907246	Not
Sna51- Hp37	*Stellifer naso*	CAETE037-20/OQ075648	*Stellifer naso*	Sciaenidae	100%	ANGBF30656/ACS2557	100%	KJ907248	Not
Sna52- Hp38	*Stellifer naso*	CAETE038-20/OQ075649	*Stellifer naso*	Sciaenidae	99.79%	ANGBF30656/ACS2557	99.79%	KJ907248	Not
Sna49- Hp39	*Stellifer naso*	CAETE039-20/OQ075650	*Stellifer microps*	Sciaenidae	99.79%	ANGBF30642/ADC2628	100%	KJ907246	**Yes**
Sna53- Hp40	*Stellifer naso*	CAETE040-20/OQ075651	*Stellifer naso*	Sciaenidae	99.79%	ANGBF30656/ACS2557	99.79%	KJ907248	Not
Cps34- Hp41	*Colomesus psittacus*	CAETE041-20/OQ075652	*Colomesus psittacus*	Tetraodontidae	100%	GBGCA5186/ACA6880	100%	KC959923	Not
Cps35- Hp42	*Colomesus psittacus*	CAETE042-20/OQ075653	*Colomesus psittacus*	Tetraodontidae	99.79%	GBGCA5186/ACA6880	99.79%	KC959923	Not
Cps37- Hp43	*Colomesus psittacus*	CAETE043-20/OQ075654	*Colomesus psittacus*	Tetraodontidae	99.79%	GBGCA5186/ACA6880	99.79%	KC959923	Not
Cps38- Hp44	*Colomesus psittacus*	CAETE044-20/OQ075655	*Colomesus psittacus*	Tetraodontidae	99.79%	GBGCA5186/ACA6880	99.79%	KC959923	Not
Ste39- Hp45	*Sphoeroides testudineus*	CAETE045-20/OQ075656	*Sphoeroides testudineus*	Tetraodontidae	100%	ANGBF25081/AAB1130	100%	KC959927	Not
Ste42- Hp46	*Sphoeroides testudineus*	CAETE046-20/OQ075657	*Sphoeroides testudineus*	Tetraodontidae	99.79%	GBMIN129531/AAB1130	99.79%	KC959927	Not

After comparing the sequences with those available in public databases, 70% of samples corroborated previous morphological identification no misidentifications. However, some incongruencies between morphological and molecular identification were verified. In general, 22% of DNA haplotypes belonged to a distinct species in relation to traditional species methods while 8% of them had no comparable species-specific sequences in public databases (Table 1).

**Fig 2:**
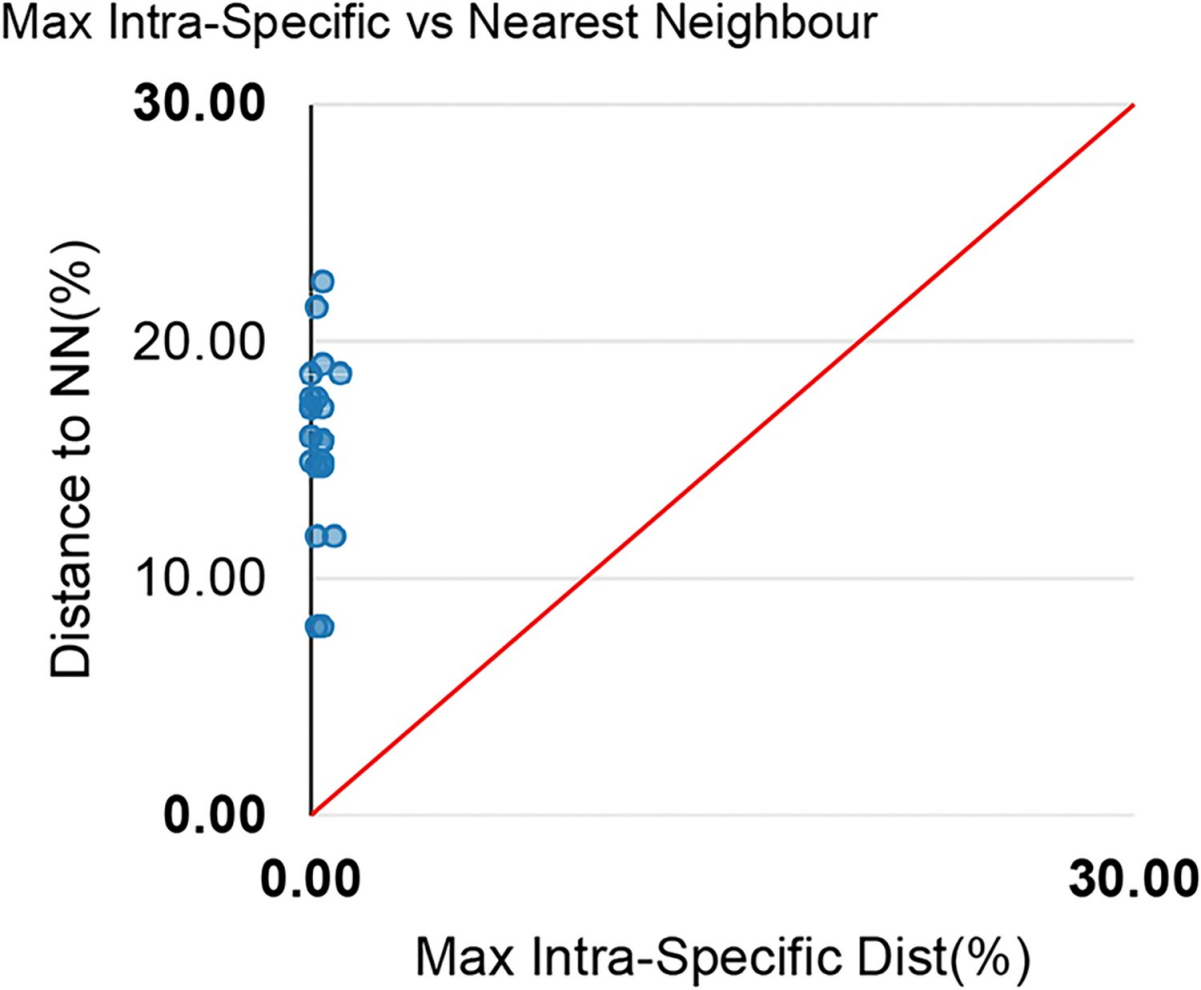
Graphic representation of maximum values of intra and interspecific distances (nearest-neighbor) for the juvenile fish of Caeté River estuary, Brazilian Blue Amazon.

**Fig 3:**
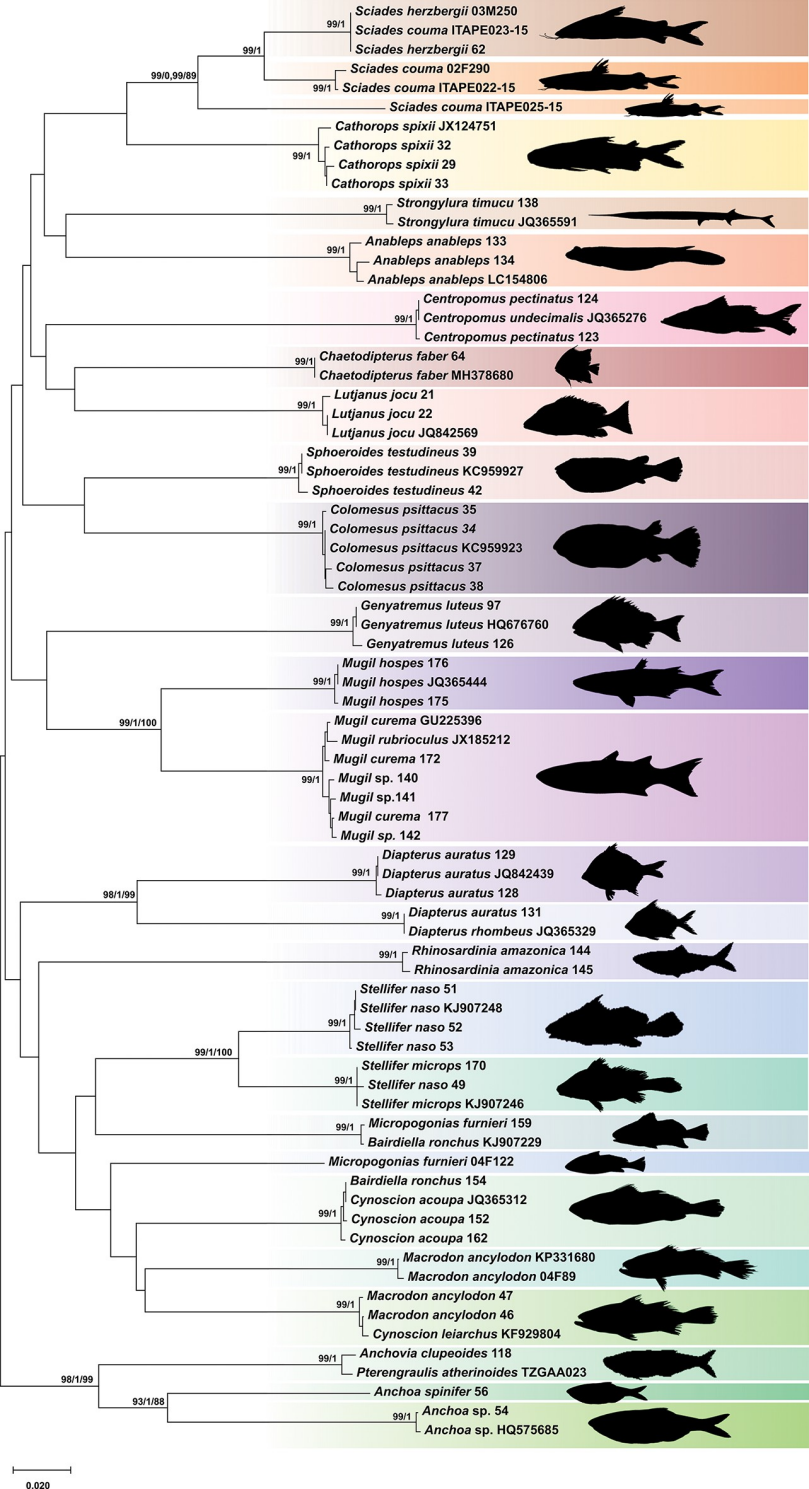
Neighbor-joining tree, showing haplotype clusters of the 74 juvenile fish specimens of Caeté River estuary. The statistical support values are shown on internodes, and they are represented from left to right in the following order: NJ = Neighbor-joining; BI = Bayesian inference; ML = Maximum likelihood. Each color indicates a discriminated species and is accompanied by their silhouette.

**Fig 4:**
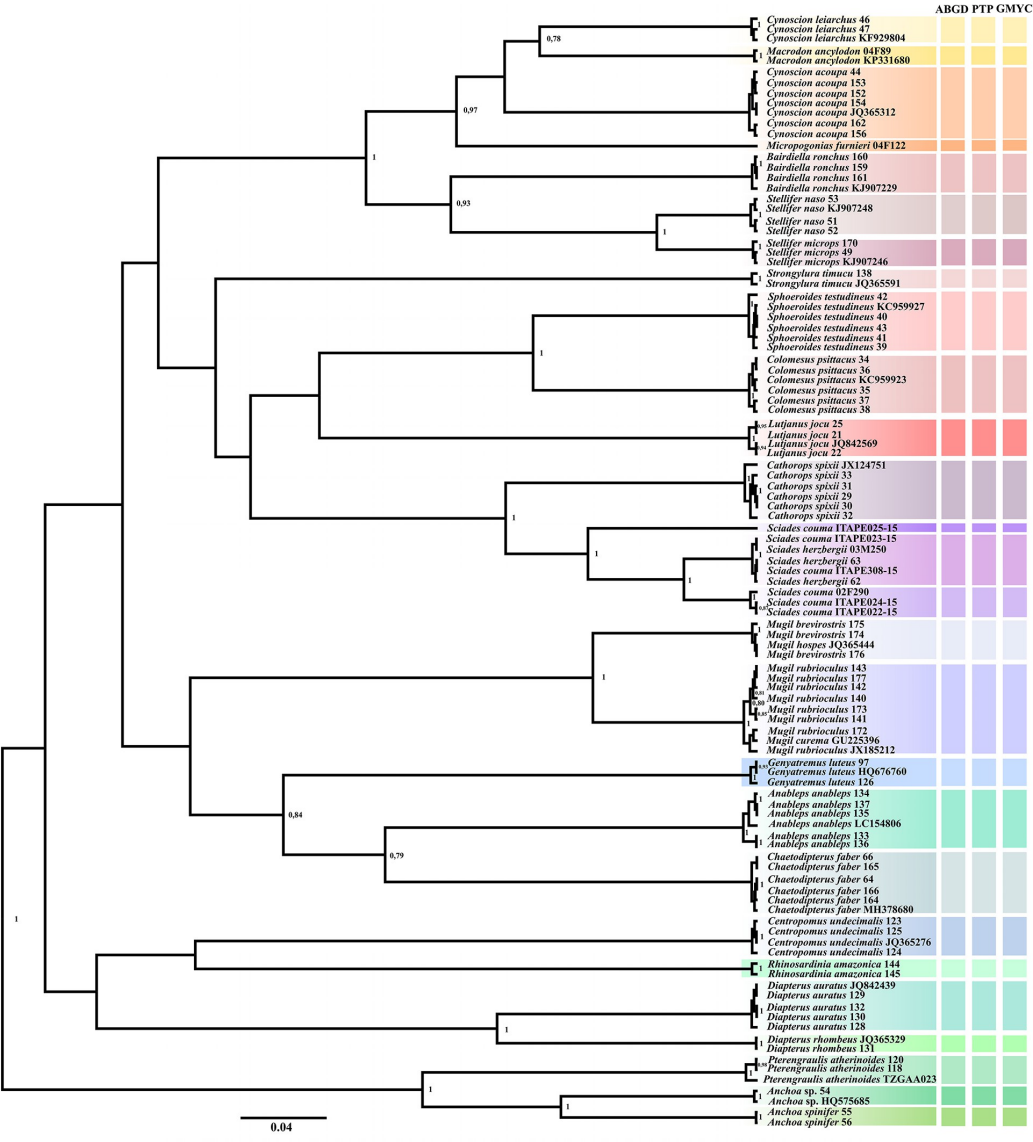
Ultrametric topology for the species delimitation scenarios in fish fauna from Caeté River estuary. The vertical bars correspond to each lineage, the colors indicate the discriminated species.

No overlapped mean values were observed between the intra and interspecific genetic distances (Fig 2), thus confirming the existence of barcode gaps. The maximum values of intraspecific distance was recorded in the largescale four-eyes *A*. *anableps* (1.07%). Similarly, all methods of phylogenetic reconstruction discriminated the sampled taxa (Fig 3), resulting in mutually monophyletic and strongly supported clades for each species. The misidentification issues based on morphological traits were evidenced by blasting the sampled sequences with public datasets and later corroborated by the phylogenetic clusters. Furthermore, the species delimitation algorithms (ABGD, PTP, GMYC) invariably recovered the same species sets indicated by phylogenetic analyses (Fig 4).

The representative of Scianidae encompassed the largest number of discrepancies in relation to morphological identification within the 12 sampled families. For instance, some individuals previously morphologically identified as *C*. *acoupa* (Cac152, Cac153, and Cac162), *B*. *ronchus* (Bro154 and Bro156) and *M*. *ancylodon* (Man44) clustered together in a single clade belonging to *C*. *acoupa* (JQ365312) with high probabilities values (100%). Taking into account the genetic distance, *C*. *acoupa* representatives misidentified as *B*. *ronchus* (Bro154 and Bro156) presented 21% of genetic divergence to public sequence of *B*. *ronchus* (KJ907229). High values (20%) were also observed between the sample previously named as *M*. *ancylodon* (Man44) and public sequences of *M*. *ancylodon* (KP331680), revealing incorrect identification based on morphology.

Two other individuals morphologically identified as *M*. *ancylodon*–Man46 and Man47, showed similarities equal or higher to 99% with *C*. *leiarchus* (KF929804), while they presented a genetic divergence of 19% when compared to DNA sequences of *M*. *ancylodon*. Moreover, the specimens identified as *M*. *furnieri–*Mfu159, Mfu160 and Mfu161, clustered with individuals of *B*. *ronchus* (KJ907229) (99%) and had a genetic mean distance of 22% in relation to *M*. *furnieri* (code 04F122) from public databases.

Similar conflicts were also noticed between the morphological and molecular identification for some congeneric species, such as in individuals identified as *S*. *naso–*Sna49, which were molecularly characterized as *S*. *microsps* (KJ907246) with a similarity value of 99%, versus 9% of divergence when compared to *S*. *naso*. Likewise, cases of misidentification were observed in the family Carangidae inasmuch as samples previously identified as *D*. *auratus–*Dau131 would actually correspond to *D*. *rhombeus* (JQ365329) (99%), while presenting a genetic divergence of 20% from *D*. *auratus* (JQ842439). Within Centropomidae, all samples initially identified as *C*. *pectinatus* (Cpe123, Cpe124, and Cpe125) proved to be 99% similar to *C*. *undecimalis* (JQ365276).

In the case of specimens of *R*. *amazonica* the barcode method could not be successfully applied because there are no other sequences available for this species in public databases. A similar scenario was observed within Engraulidae, since there are no public barcode sequences for *A*. *clupeoides* in GenBank, even though, this sample was clustered along with *P*. *atherinoides* (TZGAA023) in BOLD (99.65%). In addition, another two samples morphologically identified as *A*. *spinifer*–Asp55 and Asp56, could not be identified at species level based on molecular comparisons even though they formed a single clade in the delimitation, analyses (Fig 4). The species identified as *A*. *spinifer–*Asp54 was retrieved as *Anchoa sp*. (HQ575685) on NCBI and as *C*. *edentulus* on the BOLD system, but unfortunately this sequence was not available to add in our dataset (private access), being thus represented by two clades: (i) Asp55 and Asp56; (ii) Asp54 and *Anchoa sp*. (HQ575685).

The specimens morphologically identified as *S*. *herzbergii–*She62 and She63 were retrieved as *S*. *couma* (ITAPE023-15 and ITAPE308-15) in BOLD. However, in comparisons with the reference dataset, containing sequences of individuals of *S*. *herbergii* 03M250 and *S*. *couma* 02F290, we observed that She62 and She63 clustered along with *S*. *herbergii* 03M250. Other sequences from BOLD referred to a *S*. *couma* were included in the present analyses and divided into four clades in both NJ and BI trees.

As for the family Mugilidae, the morphological identification indicated the sampling of three species: *M*. *hopes*, *M*. *curema* and *Mugil sp*. On the other hand, the molecular discrimination evidenced two clusters: (i) *M*. *hospes* and (ii) *M*. *curema* and *Mugil sp*. The identifications after comparisons with public datasets remained ambiguous, hence the specimens named as *M*. *hospes* (Mho174, Mho175 and Mho176) were identified as *M*. *hospes* (JQ365444) on NCBI, while *M*. *hospes* (Mho175) was recovered as *M*. *brevirostris* on BOLD (private access). Similarly, samples previously identified as *M*. *curema* (Mcu172, Mcu173, Mcu177) and *Mugil* sp. (Msp140, Msp141, Msp142, and Msp143) were identified as *M*. *rubrioculus* (JX185212) and *M*. *curema* (GU225396) on NCBI, indicating inconsistencies in public sequences.

The morphological identification of the remaining samples species were confirmed by genetic data, thus showing congruent results between traditional morphology and DNA barcoding.

## Discussion

In this study, we successfully amplified barcode COI sequences from 74 juvenile fish specimens from Caeté River Estuary, located in the Brazilian Blue Amazon, a biodiversity hotspot [55] and compared them to morphological identification. Furthermore, this study also contributed with reference data for improving the datasets of public platforms, like GenBank (National Center for Biotechnology Information) and BOLD (Barcoding of Life Database), by generating 46 haplotypes of COI gene from 23 coastal fish species.

### Molecular identification: COI barcode to discriminate juvenile fish

Our results found several discrepancies between previous morphological identification and molecular data (Table 1). Accordingly, 22% out of the 46 haplotypes recovered from 74 samples encompassed morphological misidentifications, while 70% of them revealed concordant results between traditional and DNA-bases. In turn, 8% of samples could not be confirmed due to the lack of matched sequences in public datasets. The incorrect and ambiguous identification of icthyofauna is a major issue to reliable estimates of regional biodiversity and to management of stocks in fisheries [10]. These obstacles are particularly noticed in juvenile fish since most diagnostic characters are presented only on adults, as presently reported. As a matter of fact, taxonomic keys of fish identification that include all development stages, like eggs, larvae, juveniles and adults are very scarce and difficult to achieve, once each species undergoes unique morphological changes, hindering the compilation of all these stages into a single identification key [55]. To overcome this issue, taxonomic analyses that could be carried out independently on their ontogenetic development state are recommended. Otherwise, identification based on distinct stages of life should be more conservative, focused on family or genus level, even though misidentification based on morphological traits were observed even at higher taxonomic levels.

**Fig 5:**
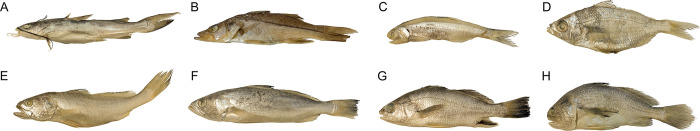
Fauna of juvenile fish from the Caeté River, Bragança-PA, Blue Amazon, that exhibited morphological identification errors. A: *Sciades couma* (total length = 9,7 cm). B: *Centropomus undecimalis* (total length = 8,8 cm). C: *Pterengraulis atherinoides* (total length = 5,4 cm). D: *Diapterus rhombeus* (total length = 7,6 cm). E: *Cynoscion acoupa* (total length = 12,2 cm). F: *Cynoscion leiarchus* (total length = 11,6 cm). G: *Bairdiella ronchus* (total length = 10,1 cm). H: *Stellifer microps* (total length = 9,7 cm).

In spite of identification errors based on morphology, all inconsistencies could be resolved by combining DNA-based phylogenetic trees and species delimitation algorithms. Overall, the highest number of erroneous previous identification was reported within the family Sciaenidae, probably related to limitations of identification at species level based only on morphology of juvenile individuals of several fish groups (Fig 5). In fact, the lack of reliable diagnostic anatomic features are well known in representatives of Scianidae, such as the genera *Stellifer* vs. *Bairdiella*, and *Cynoscion* vs. *Macrodron* [33, 56–58] (Fig 5).

Similar taxonomic issues are observed in the family Centropomidae because of their remarkable conservative morphology, as presently reported in samples morphologically identified as *C*. *pectinatus* that actually corresponded to *C*. *undecimalis* [59] (Fig 5). In Table 2, we highlight the main morphological characteristics that contributed to the misidentification of the analyzed specimens. Therefore, the association of molecular tools and morphological identification should be a common practice in the characterization of juvenile fish specimens, especially focused on species identification sharing similar anatomical traits.

**Table 2:** List of morphological characteristics that contributed to the incorrect identification of juvenile fish fauna in the Caeté River, Bragança-PA, Blue Amazon.

Morphological identification	Identified species	Family	Morphological characters
*S*. *herzbergii*	*S*. *couma*	Ariidae	Similar shape of the nucal plate between both species at juvenile stage.
*C*. *pectinatus*	*C*. *undecimalis*	Centropomidae	Overlapping characters between the two species in the count of scales above the lateral line and the count of pores on the lateral line (up to the base of the caudal fin); difficult counting of gill rakers on the first arch.
*A*. *clupeoides*	*P*. *atherinoides*	Engraulidae	Overlapping characters between the two species, such as the same count of dorsal and anal spines and a short snout; *A*. *clupeoides* exhibits a silver stripe that disappears through the ontogenetic development, making it difficult to distinguish between bothspecies.
*D*. *auratus*	*D*. *rhombeus*	Gerreidae	Difficult counting of rays in the anal fin; character of body depth relative to standard size difficult to distinguish between bothspecies.
*B*. *ronchus*	*C*. *acoupa*	Sciaenidae	Overlapping characters in the count of spines in the dorsal fin and rays in the anal fin; similar pelvic fins between both species; distinctive coloration in *C*. *acoupa* not present in juveniles.
*M*. *ancylodon*	*C*. *leiarchus*	Sciaenidae	Difficult counting of spines and rays in the dorsal and anal fins in juveniles.
*M*. *furnieri*	*B*. *ronchus*	Sciaenidae	Overlapping characters in the count of rays in the posterior portion of the dorsal fin and rays in the anal fin; pattern of dark stripes below the lateral line present in both species.
*S*. *naso*	*S*. *microps*	Sciaenidae	Overlapping characters in the count of rays in the dorsal fin and gill rakers on the first arch; in addition to subjective characters to identify *S*. *microps*, such as mouth size and body coloration.

### Incongruencies in public sequence databases

When the presently obtained sequences were compared to those from public datasets (BOLD and NCBI), we detected ambiguities in some groups, such as Mugilidae and Ariidae. In the former, the COI sequences identified as belonging to *M*. *hospes* shared similarity values between 99.79% and 100% with public sequences available for the same species in NCBI. However, in a recent revision, the occurrence of *M*. *hospes* in Southern Atlantic was refuted, while *M*. *brevirostris* was recognized [60]. Thus, the sequences of Brazilian samples identified as *M*. *hospes* in GenBank should actually correspond to *M*. *brevirostris* [61].

In addition, the sequences from *Mugil sp*. and *M*. *curema* blasted with COI sequences from *M*. *curema* and *M*. *rubrioculus* available in NCBI (Table 1). As a matter of fact, uncertainties in the species identification of public sequences of *Mugil* are frequent, most likely because of the lack of morphological diagnostic traits related to the high occurrence of cryptic species complexes in this genus [61]. Therefore, a thorough revision of stored sequences in public databases is recommended, as well as a continuous upload of COI sequences of Mugilidae from distinct regions.

Similarly to Mugilidae, inconsistencies in the identification of *Sciades* (Ariidae) among the available COI data possibly resulted from inaccurate morphological identification of samples. It should be pointed out that the samples of this genus, which included reference sequences of two adult individuals (*S*. *herzbergii–*She03M250 and *S*. *couma–*Sco02F290) were divided into three clusters in both phylogenetic trees (Fig 3) and species delimitation methods (Fig 4). Therefore, our samples would probably comprise three species, *S*. *couma*, *S*. *herzbergii* and a third one, which we believe belong to another genus within Ariidae. The divergence values among them varied from 6% (She03M250 x Sco02F290), 12% (*S*. *couma* ITAPE025-46 x She03M250) and 13% (*S*. *couma* ITAPE025-46 and Sco02F290).

Ambiguous deposits in public BOLD and NCBI systems have been widely reported and debated in the literature [61]. Thus, researchers should proceed with extra caution during validation of species identification, including reference to updated literature and assistance from experts in taxonomy of the selected organisms. This course of action is particularly important in studies of ichthyofauna, a remarkable diversified group [61]. In parallel, we strongly encourage continuous revisions of public sequences to solve ambiguous cases, mitigate errors and to assure their reliability.

In the case of Engraulidae, three clusters of distinct taxa were observed. The sample identified as *Anchoa sp*. Blasted with *A*. *spinifer* clade 54 in NCBI, while in BOLD it presented 100% of similarity with *C*. *edentulus*, a species distributed from the Caribbean and Panama up to the coast of state of Santa Catarina, in southern Brazil [62]. Moreover, *C*. *edentulus* is commonly found in estuarine environments [63]. On the other hand, the specimens morphologically identified as *A*. *clupeoides* were subsequently identified as *P*. *atherinoides* according to 99.15% of similarity with COI data available in BOLD.

The family Engraulidae is a monophyletic group, currently divided into two major subfamilies: Coilinae and Engraulinae. The latter is composed of two clades, referring to marine and freshwater taxa, respectively [64]. All freshwater taxa in Engraulinae are derived from a single event of marine transition in New World [64]. On the other hand, no matches were observed for the haplotype *A*. *spinifer* 56, which was recovered into a distinct clade, thus probably representing another congeneric species. In addition, the number of open access sequences of Engraulidae should be increased in public databases. For instance, the available sequences of *C*. *edentulus* remains private, thereby hindering their utilization in present analyses. Furthermore, the only representative of Clupeidae sampled in the present work, *R*. *amazonica*, lacked previous COI data in public datasets.

The matches between public sequences referred to as *D*. *rhombeus* and *D*. *auratus–* 131 haplotype illustrates the difficulties in distinguishing many species of the family Gerreidae. This can be explained by the fact that morphological key identifications for this fish group is highly subjective and associated with a high number of overlapped meristic and morphometric traits [65, 66].

### Effectiveness of DNA barcoding

Fish are the diverse group of vertebrates, presenting several morphological adaptations to their ecosystems. As observed in our analyses, these traits are usually overlapped among distinct taxa, thus limiting precise taxonomic inferences based only on anatomical features, particularly in biodiversity hotspots [67].

Furthermore, traditional taxonomy, which relies only on morphological analysis, has often failed in identifying juveniles or of closely related and morphologically similar species, as reported in other studies [20, 68–70]. Our investigation validated the efficacy of DNA barcoding to discriminate species [5] from highly diversified habitats like the Caeté River estuary, which is part of coastal zone in Brazilian Amazon [71]. Most issues were attributed to the phenotypic similarities of species, especially when juveniles [7, 20]. In addition, some taxa lacked reference sequences in public platforms such as BOLD and NCBI. The intraspecific mean distance and the nearest neighbor-joining analyses (NJ) provided by BOLD workbench confirmed the efficiency of DNA barcoding in the species-specific discrimination of samples. Both the maximum and the mean intraspecific distance values (1.07% and 0.24%, respectively) were below than the NN distances (maximum of 22.53%, minimum of 7.98% and a mean value of 15.95%), without overlapped values (Fig 2). As previously described, the maximum intraspecific distance mean value was 1.07% observed in *A*. *anableps*, which might comprehend two sympatric species, as previously reported in Caeté River [72].

In the present study, we generated the first molecular characterization of fishes from the Caeté River by DNA barcoding. However, the use of this molecular tool requires specialized facilities and costs, which unfortunately are out of reach for many research and conservation centers. Thus, we suggest the use of DNA barcode as a support for integrative identification and taxonomy focusing on studies of fish diversity, especially in groups that remain a challenge for traditional approaches, such as juvenile specimens. Furthermore, we encourage a continuous uploading of open access sequences with confirmed species identification in public genetic datasets, as well as a thorough revision of available sequences.

## Conclusions

This study corroborates the efficiency of the DNA barcoding to accurately identify fish species. The use of this molecular marker was efficient to discriminate the juvenile fish fauna from Caeté River estuary, placing this approach as a suitable diagnostic tool, particularly in cases where traditional morphologically-based identification fails. In the current scenario of several overexploited species threatened to extinction, conservation programs need to be fostered. Once the utilization of DNA barcodes are successfully established for the identification of species, as presently demonstrated, a combined strategy based on traditional and DNA barcode identification would be essential to achieve both scientific and practical benefits in evaluating the sustainability and management of fisheries resources.
